# Beyond Distracted Eating: Cognitive Distraction Downregulates Odor Pleasantness and Interacts with Weight Status

**DOI:** 10.3390/nu16172871

**Published:** 2024-08-27

**Authors:** Iryna Ruda, Deepak Charles Chellapandian, Marlene Rott, Selina Scheid, Jessica Freiherr

**Affiliations:** 1 Department of Psychiatry and Psychotherapy, Friedrich-Alexander-Universität Erlangen-Nürnberg, Schwabachanlage 6, 91054 Erlangen, Germany; 2 Sensory Analytics and Technologies, Fraunhofer Institute for Process Engineering and Packaging IVV, Giggenhauser Strasse 35, 85354 Freising, Germany

**Keywords:** distracted eating, odor perception, odor intensity, odor pleasantness, obesity, hedonic eating

## Abstract

Considering the widespread issue of distracted eating, our study investigates how cognitive distraction influences the sensory perception of food-related odors among individuals with varying weight statuses. We conducted an exploratory, randomized, and cross-sectional experimental study, using the Tetris game to simulate real-life cognitive distraction, incorporating two distraction levels (low and high) and presenting five distinct odors. A total of 59 participants, categorized into a lean (*n* = 30) and overweight/obese group (*n* = 29) based on their body mass index (BMI), received odor stimuli while playing Tetris at low and high difficulty, corresponding to low and high distraction levels, respectively. Participants subsequently rated odor intensity and pleasantness under the two cognitive distraction conditions. Respiratory movements were monitored to ensure accurate olfactory stimulation. Our findings revealed no significant difference in odor intensity ratings across distraction levels (*p* = 0.903). However, there was a significant reduction in odor pleasantness under high cognitive distraction (*p* = 0.007), more pronounced in lean participants compared to those with an overweight status (*p* = 0.035). Additionally, an interaction between gender and cognitive distraction effects was observed in odor pleasantness perception. The differential effects of distraction across weight-status groups and genders are discussed in the context of hedonic motivation and compensatory mechanisms. This study sheds light onto the sensory mechanisms underlying distracted eating and could inform more personalized strategies for promoting healthier eating habits in a world dominated by distractions.

## 1. Introduction

In our fast-paced society, distracted eating—with meals being consumed in parallel with other activities—is exceedingly prevalent [1,2]. Eating in distraction-rich environments, like watching TV [3,4,5], being in front of other screens [6,7,8], or while driving [9], is linked to overconsumption and a higher body mass index (BMI). It is thus obvious to claim distracted eating as a contributing factor to the current obesity pandemic [10,11].

Several mechanisms have been proposed to explain why distracted eating leads to overconsumption. It is evident that attention that is drawn away from eating impedes the ability to adequately monitor food intake and hinders the perception of internal signals of fullness, resulting in overeating [12]. Moreover, distraction during eating downregulates the intensity perception of food flavor, potentially prompting overeating and altering preferences towards more palatable high-caloric foods. It is therefore essential to elucidate how distraction affects the sensory aspects of food, as this provides valuable insights into eating behavior and broader sensory interactions with our environment.

While flavor perception—an overall sensory experience of what we consume—is a complex interplay of all sensory modalities, odor perception is of pivotal significance in general and is of interest particularly for this study [13]. In fact, well before food is consumed, odor perception forms the anticipation of food intake, stimulates appetite, and also shapes eating preferences and overall eating experience [14,15]. Furthermore, odors can impact mood and affective states, which in turn influence dietary choices [16,17]. Interestingly, even odors perceived unconsciously or non-attentively have potent power to impact food choices [18]. Odor perception is mainly characterized by hedonic valence, intensity, and quality, features that collectively shape our olfactory experiences and interactions with our environment.

Prior evidence, primarily from our lab, demonstrates that odor perception, particularly odor intensity, is significantly affected by cognitive distraction. Using a memory task as a cognitive load paradigm, we found that a reduced perception of odor intensity observed under high cognitive load was also evident at the neural level. Utilizing functional magnetic resonance imaging (fMRI), we showed that distraction reduces activation in brain regions responsible for olfactory processing, including the piriform cortex, orbitofrontal cortex, and insula [19]. A follow-up behavioral study by Schadll et al. (2021) replicated the observed decrease in odor intensity perception under high cognitive load, involving a more realistic cognitive load paradigm, the Tetris game [20].

Despite the global rise in obesity rates, research on distracted sensory perception has predominately focused on individuals of normal weight and mainly females [21,22,23,24]. This is disappointing, as individuals with an overweight or obesity status constitute approximately 43% and 16% of the world’s population, respectively [25]. As we strive to promote healthier eating habits on a global scale, it is crucial to understand the impact of distraction on smell perception in diverse populations, taking weight status and gender into consideration.

In the current study, we also utilized the Tetris game as a cognitive task comprising low and high levels of distraction, since it offers a more realistic experience mimicking the dynamic cognitive distractions encountered in everyday life situations [26]. Moreover, the engaging nature of the Tetris game enables longer experimental duration without inducing boredom [27,28]. To capture how sensory performance changes under distraction, we contrasted intensity and pleasantness ratings obtained in low and high cognitive distraction conditions. We then systematically compared the differences across conditions in participants with excessive weight and obesity to participants of normal weight across different genders. Importantly, breathing patterns are known to influence odor perception [29]. Therefore, we accounted for breathing variabilities when exploring effects of cognitive distraction on intensity and pleasantness ratings.

With regards to odor intensity, we hypothesized that cognitive distraction may have a greater influence in individuals with obesity, resulting in a more profound decrease in odor intensity perception. The rationale behind this is that obesity has been linked to diminished odor perception [30], impaired inhibitory control [31], and altered food reward processing [32].

Often, the hedonic valence—the degree to which an odor is perceived as pleasant or unpleasant—is a dominating if not the first and most important characteristic of an odor [33], greatly determining food choices but not limited to eating behavior. Pleasantness of odors is intricately linked to the reward system and especially relevant in the context of hedonic eating, prevalent in obesity [34,35]. To our knowledge, no studies have investigated how the hedonic valence of odors changes with increased distraction. This study was designed to answer this research question. Given the lack of prior research and the complex interplay between odor pleasantness perception and eating behavior, weight status, and reward-driven choices, we hypothesized that changes in pleasantness perception under distraction would vary between participants of different weight statuses. However, we did not specify whether these changes would manifest as an increase or decrease in pleasantness perception.

The goal of this study was to provide novel insights on the effects of cognitive distraction on the intensity and pleasantness perception of odors, highlighting the role of weight status and gender, while addressing the raised limitations of the prior studies.

## 2. Materials and Methods

To investigate the impact of cognitive distraction on olfactory performance, we conducted an exploratory, randomized, and cross-sectional experimental study, using two distraction levels and presenting five distinct odors to two different weight-status groups.

This study was approved by the ethics committee of the University Hospital Erlangen (project number: 128_21 B) and complied with the revised Declaration of Helsinki. This study is a part of a larger ongoing study exploring the effects of cognitive distraction on chemosensory perception and processing across volunteers of various weight statuses.

### 2.1. Participants

Participants were recruited via flyer distribution, online advertisements, and word of mouth. When declaring interest in this study, participants were provided with a personal login code and the access link to the online questionnaire screening for eligibility criteria. Participation in this study was granted when meeting the following criteria: (1) a body mass index (BMI) between 18.5 and 40 kg/m^2^; (2) scoring less than 19 on the Beck Depression Inventory (BDI, [36]); (3) no pregnancy or breastfeeding; (4) no known, untreated, thyroid dysfunction; (5) no respiratory diseases; (6) no chronic metabolic or neurological diseases; (7) no smoking. Selected inclusion criteria are commonly used in chemosensory research to minimize variables that could affect smell perception, ensuring more accurate and reliable results. We set a BMI cut-off of 40 kg/m^2^ to avoid including volunteers with medical conditions (e.g., metabolic syndrome or a history of bariatric surgery), which could introduce variables and affect comparability. Overall, 34 volunteers were excluded after completing the online questionnaire and the remaining 62 participants were invited to the University Hospital Erlangen for the experimental session.

On the day of the experiment, participants’ BMI was measured and calculated as body weight (kg)/square of body height (m^2^) and, if required, self-reported values were corrected. To confirm normosmia of all participants, on site, we performed the 16-item Sniffin’ Sticks identification (SSI) test [37]. If a participant identified less than 11 out of 16 odors correctly, their participation in this study was terminated. In total, 3 participants were excluded due to failing the SSI. Thus, the final study sample was composed of 59 participants (lean: *n* = 30, obese/overweight: *n* = 29). All participants signed a written informed consent form and data security statement prior to participation in the experiment.

Subjects were asked not to eat anything, not to smoke tobacco, and to drink only water one hour before the experiment to avoid various influences on the olfactory system. Before the experiment, the participants were informed verbally and in writing about the study procedure and that they have the right to withdraw from this study at any moment. After the completion of this study, all participants were monetarily compensated.

Following the definition provided by the World Health Organization, the participants with 18.5 ≤ BMI ≤ 24.9 kg/m^2^ would form a lean group and participants with a BMI range from 25 to 40 kg/m^2^ would be assigned to the overweight/obese group. In other words, participants with excess weight and those classified as individuals with obesity were pooled together in one group. The formed groups were only relevant for analysis purposes. In this manuscript, we define “gender” as a binary construct, including both biological sex and individual self-identification. This definition is based on the fact that all participants identified themselves as either male or female when presented with additional options (“non-binary”, “diverse”, “your option”).

### 2.2. Olfactory Stimulation

During the preparation phase before this study, eleven volunteers—who did not participate in the main study—selected four out of eight food-associated odors that were perceived as the most edible and covered a broad range of pleasantness. All odor stimuli (but not coffee) were diluted with propylene glycol and concentrations were calibrated to achieve similar perceived intensity. The final stimulus pool consisted of these odors: beef (Symrise AG, Holzminden, Germany; beef aroma type cooked, 10%), cheese (Symrise AG, Holzminden, Germany, pizza aroma type Margherita, 7.5%), coffee (Edeka Zentrale Stiftung & Co. KG, Hamburg, Germany, powder, 100% Arabica), mango (CPL Aromas Ltd., Bishop’s Stortford, UK, Mango AR681772, 10%), and an odorless (empty) stimulus represented by pure propylene glycol. The implementation of the odorless condition within this study served a double purpose: enabling olfactory receptors “to rest” between the odorous stimuli to create an effective, yet comfortable, experience for the participants and serving as a control condition. Odors were stored in the refrigerator and brought to room temperature at least an hour before the actual experiment started to ensure equal smell quality for each testing. A computer-controlled olfactometer was set up for odor delivery, producing a constant air flow of 3 L per minute [38]. Constant air flow was delivered to the participants across the entire experiment with odor stimulation embedded into it. Each of five odors was delivered simultaneously to both nostrils in a block design manner with 2 s of stimulation and 4 s of rest. In each trial, only one odor was presented four times in the 24 s that formed a sensory stimulation window.

### 2.3. Procedure

This study employed the Tetris game paradigm to serve as the cognitive distraction framework [20]. The Tetris game is a puzzle video game where players arrange falling geometric shapes to form complete horizontal lines, which then disappear. The goal is to clear as many lines as possible before the playing field fills up [39]. In our study, we introduced two levels of cognitive distraction, categorized as low and high, which were created by varying the speed of falling blocks in the game. Olfactory stimulation took place simultaneously with Tetris and participants were asked to rate odor intensity and pleasantness under low and high distraction conditions.

The experimental session lasted around 50 min and consisted of two parts: the practice part followed by the main experiment. The practice part was implemented to accustom subjects to the odor’s variability and the application setup and ensure the knowledge of the controls of the Tetris game. Questions were allowed at any point during this part. In this part, each stimulus was presented twice for 3.5 s. After each odor presentation, subjects provided a rating of perceived intensity and pleasantness of the stimulus. Participants were informed that there might be trials where they would not smell anything, referring to the trials with the odorless stimulus. After the last odor presentation, participants saw the instructions for the Tetris game on a screen and played a brief introduction trial to get used to the key bindings.

In the following experimental part, participants were instructed to play the Tetris game to the best of their ability and to evaluate how intense and how pleasant they perceived an odor to be as well as how difficult each Tetris game trial was. A 100 mm visual analog scale was utilized for each of the ratings with the left (lowest) anchor labeled as “not at all” and the right (highest) labeled as “very”. To confirm that our cognitive distraction paradigm effectively created two distinct levels of cognitive load, we monitored Tetris game performance and recorded the reported difficulty of each trial. The number of rows successfully cleared in each Tetris trial—Tetris scores—provided objective information whether the game performance dropped during the high cognitive load condition. Meanwhile, difficulty ratings contributed to the subjective perception of cognitive load manipulation. Note that participants were not aware of the real focus of this study to not bias their responses.

**Figure 1:**
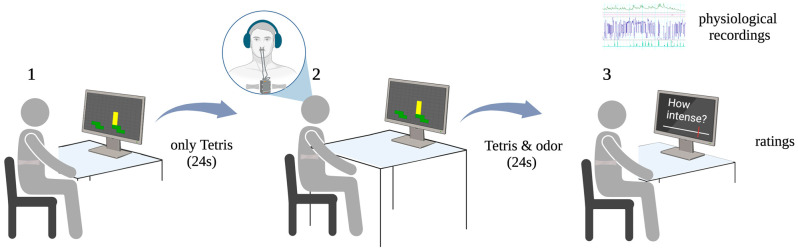
Graphical depiction of a trial. 1. The participant plays the Tetris game. 2. The participant continues to play the Tetris game while an odor is being delivered via the setup demonstrated in a magnified circle. 3. After 48 s of the Tetris game the participant provides the ratings on intensity, pleasantness, and difficulty. For the whole duration of the experiment, respiratory data and skin conductance data are recorded. Created with BioRender.com.

In total, there were 40 trials in the experiment, with each lasting 48 s (see Figure 1). During each trial, participants first played the Tetris game with no odor stimulation for 24 s and then another 24 s with stimuli applied in the manner described earlier. Such a design assured the immersion effect of the Tetris game. When ready, participants could press a button to start a new trial. During the experiment, each odor was presented during eight trials, four times in a low cognitive load condition and four times in a high cognitive load condition. The odor presentation was randomized with no direct repetitions of the same smell modality in two consequent trials.

Additionally, to elucidate potential effects of satiety on perceived odors, we assessed participants’ hunger level by means of the 100 mm visual analog scale at the various times during the experiment: in the beginning of the practice session, in the middle, and after the last trial of the experiment.

For the whole duration of the experiment, participants wore headphones playing white noise to mask the noise of stimuli delivery and the surrounding noises to avoid unprompted distraction. The experiment was designed and executed using PsychoPy3 software [40]. A keyboard was used to navigate the Tetris game.

### 2.4. Physiological Measurements

Together with the behavioral performance, the physiological parameters such as breathing patterns and skin conductance response (SCR) were recorded during this study. To detect possible variations in breathing patterns between low and high cognitive load conditions, which may affect the perception of the stimuli and impact the ratings participants provide for each odor, participants wore a respiratory belt transducer (ADInstruments Ltd., Oxford, UK), which measured respiratory movements. Numerous research studies have shown that high cognitive load induces physiological alterations, which can be detected by analyzing changes in skin conductance response. Thus, with two electrodes (LT118F GSR Finger Electrodes, ADInstruments Ltd., Oxford, UK) placed on the palmar surface on the middle phalanx of the index and middle finger of the left hand, we recorded SCR. We anticipated observing higher skin conductance response values during high compared to low cognitive load conditions. All physiological data were recorded via Labchart (v8.1.25 licensed on 21 March 2023, ADInstruments Ltd., Oxford, UK) software and by PowerLab model 4/26 with FE116 GSR Amp (ADInstruments Ltd., Oxford, UK).

### 2.5. Statistical Analysis and Physiological Data Processing

Behavioral data were analyzed with the software JASP, version 0.17.2.1 (JASP Team (2024)). To calculate effects of cognitive distraction, linear mixed models were computed. Multiple comparison correction was applied to the *p*-values using the Bonferroni method. Corrected estimates and confidence intervals are reported throughout this manuscript. The odorless stimuli were not included into the stimulus pool for the analysis unless specified otherwise.

We computed a linear mixed model on the Tetris scores and one on difficulty perception with fixed factor cognitive distraction (low, high) and the random factor of participants for difficulty ratings and Tetris scores separately. To investigate a potential link between provided intensity and pleasantness ratings and the BMI, we ran a correlation analysis using Pearson´s coefficient. A dependent *t*-test was conducted on satiety ratings obtained in the beginning, in the middle, and at the end of the experimental session. The ⍺-level for all tests was set to 0.05.

For the physiological data analysis, precise time of each experimental event was used to extract the SCR signal and respiration data in an event-related fashion. A Python-based program was written to allow reading files with physiological data recorded by LabChart software (v8.1.25 licensed on 21 March 2023, ADInstruments Ltd., Oxford, UK). Then, these physiological data were converted to the generalized biosignal. Using an open-source Python package version 3.8, NeuroKit2 version 0.2.0 [41], we further processed the SCR to phasic and tonic components; the latter being used for the analysis. With regards to the respiratory signal, we first cleaned the data to identify peaks, which allowed us to determine the respiratory rate. By counting these peaks over a one-minute period, we obtained breaths per minute (BPMs), which was then used in our analysis. Physiological data of two participants were excluded due to a technical issue during the experiment, preventing data from being correctly recorded. An outlier identification analysis by visual inspection using Box plot graphs and a high-pass filter set at 30 breaths per minute led to the exclusion of 201 trials (8.51%).

## 3. Results

### 3.1. Descriptive Characteristics of the Study Sample

Data from 59 participants, categorized into lean and overweight/obese groups based on their body mass index (BMI), were analyzed for this study (see Table 1).

### 3.2. Distraction Induces a Reduction in Pleasantness Perception

**Figure 2:**
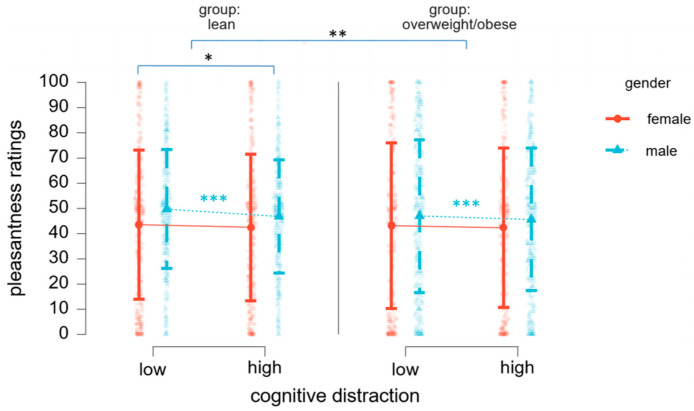
Distraction-related decrease in pleasantness rating is shown across weight statuses. Gender-related changes are color-coded. * *p* < 0.05, ** *p* < 0.01, *** *p* < 0.001.

To test our hypothesis that cognitive distraction might influence the hedonic perception of food-related odors, we computed a linear mixed model on pleasantness ratings with the fixed factors cognitive distraction (low, high), gender (female, male), group (lean, overweight/obese), and odor (beef, cheese, coffee, and mango) and a random factor of participants. Results reveal that cognitive distraction significantly reduced pleasantness perception and its effects were modulated by gender and weight status (see Table 2 and Figure 2).

When averaged across both levels of cognitive distraction, ratings of odor pleasantness did not vary based on the factors gender [*F* (1, 55.01) = 2.62, *p* = 0.110] or group [*F* (1, 55.01) = 0.07, *p* = 0.78]. We, however, observed an interaction effect of group x odor [*F* (1, 55.0) = 5.70, *p* = 0.002]. A closer look revealed that beef odor (Mean Difference = 13.49, t = 6.25, *p* < 0.001) and cheese odor (Mean Difference = 8.73, *t* = 4.06, *p* = 0.001) were perceived as more pleasant by participants in the lean group, while coffee odor was found to be more pleasant by the participants in the overweight/obese group (Mean Difference = 17.19, *t* = 7.99, *p* < 0.001). Pleasantness ratings of mango odor did not differ across groups (*p* = 1.00). A two-way interaction of gender x odor also had an effect on pleasant ratings, such that female participants liked cheese odor (Mean Difference = −12.19, *t* = −5.55, *p* < 0.001) sufficiently less when compared to the male participants.

As expected, pleasantness ratings varied significantly across odor modalities (averaged across groups, gender, and cognitive distraction; see Table 3).

Results revealed that pleasantness ratings did not vary across high (*M* = 44.74, *SD* = 17.77) and low (*M* = 44.83, *SD* = 19.02) levels of cognitive distraction [*F* (1, 464) = 0.024, *p* = 0.877).

To further examine how variability in respiration could impact odor pleasantness perception, we computed a linear mixed model with the same fixed factors of cognitive distraction, gender, group, and odor, additionally including breathing rate per minute as a factor. Results confirmed a main effect of cognitive distraction [*F* (1, 73.59) = 5.01, *p* = 0.028] and odor [*F* (3, 44.27) = 14.22, *p* < 0.001] and no effect of the group [*F* (1, 55.01) = 2.62, *p* = 0.110] on pleasantness ratings. The same interaction effect between group x odor was noticeable [*F* (1, 55.01) = 2.62, *p* = 0.041].

This time, however, results implied that pleasantness ratings were significantly impacted by gender [F (1, 44.67) = 2.99, *p* = 0.041]; in particular, female participants perceived odors as less pleasant (*M* = 41.09, *SD* = 32.69) when compared to male participants (*M* = 47.14, *SD* = 28.31). Moreover, the effect of the respiration rate on pleasantness ratings reached significance [*F* (1, 46.31) = 11.40, *p* = 0.001], elucidating that participants took more breaths per minute when odors were of higher perceived pleasantness. This observation has been further confirmed by the correlation analysis, in which results show a weak positive correlation between pleasantness ratings and breathing rate (*r* = 0.075, *p* < 0.001).

### 3.3. Effects of Distraction on Intensity Perception Interact with Gender and Breathing Patterns

To elucidate impact of cognitive distraction on odor intensity perception, we computed a linear mixed model on intensity ratings with the fixed factors cognitive distraction (low, high), odor (beef, cheese, coffee, mango), group (lean, overweight/obese), and gender (female, male), and the random factor of participants. Results indicated no significant main effect of cognitive distraction [*F* (1, 0.27) = 0.013, *p* = 0.903] or group [*F* (1, 55.03) = 1.99, *p* = 0.164]. We established a statistical trend for a gender effect influencing intensity ratings [*F* (1, 55.03) = 3.11, *p* = 0.075]. On average, female participants rated odors as more intense (*M* = 70.10, *SD* = 23.09) compared to male participants (*M* = 63.26, *SD* = 22.36). The odor modality had a significant effect on intensity perception [*F* (3, 55.03) = 19.35, *p* < 0.001] (see Table 4).

**Figure 3:**
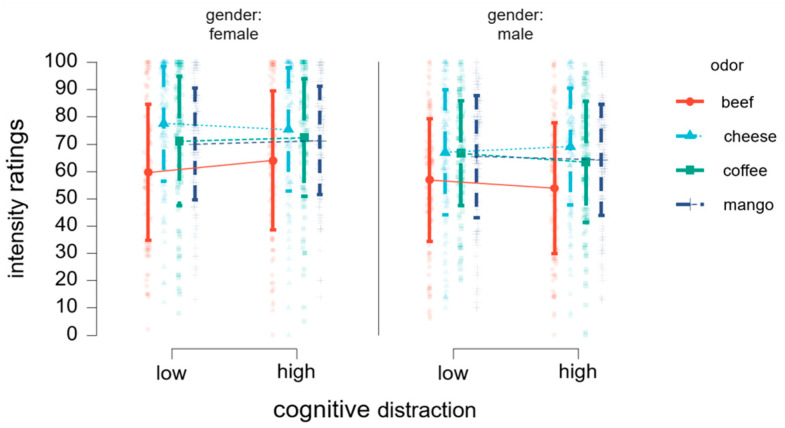
Changes in intensity ratings between cognitive load conditions (low, high). Intensity ratings from female and male participants are depicted in separate columns. Horizontal lines depict responses for each odor stimulus. An opposite distraction-related effect across genders is depicted.

There was a significant interaction effect of odor x cognitive distraction x gender [*F* (3, 102.79) = 2.79, *p* = 0.044]. Further inspection revealed a crossing interaction, with intensity perception going opposite directions between genders (see Figure 3). Yet, no significant differences were observed between any of the pairs during the post hoc analysis.

As a next step, we investigated how breathing patterns contribute to the changes in intensity perception. We thus included the breathing rate per minute (BPM) as an additional factor to the above-mentioned linear mixed model computed on intensity ratings. Its inclusion did not affect the impact of the odor, gender, and group on intensity perception, leaving all statistical results unchanged. There was no main effect of the breathing rate on intensity perception [*F* (1, 20.60) = 1.34, *p* = 0.266], but the effect of cognitive load became evident [*F* (1, 78.70) = 4.34, *p* = 0.040].

**Figure 4:**
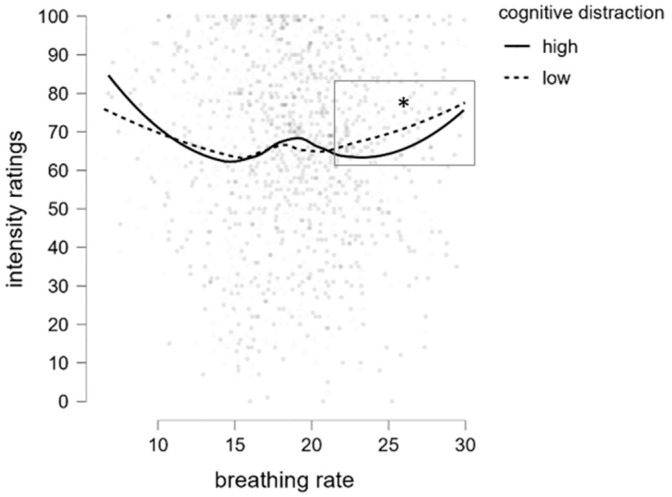
Changes in intensity ratings related to the number of breaths per minute (BPMs). Values across low and high cognitive load are represented as separate lines. A decrease in intensity perception during a high level of cognitive load in the BPM range of 22–30 is highlighted. * *p* < 0.056.

However, we also observed an interaction effect of cognitive load x breathing rate [*F* (1, 89.11) = 4.83, *p* = 0.030]. Resolving the interaction revealed that a moderate effect of cognitive load was only visible in trials with the highest breathing rate range (22–30 breaths per minute) (Figure 4). Specifically, intensity perception decreased in the high cognitive load condition (*M* = 64.72, *SD* = 22.14) compared to the low cognitive load condition (*M* = 67.79, *SD* = 20.90, Mean Difference = −3.34, *t* = −1.91, *p_Bonf_.* = 0.056) while the breathing rate did not vary between low (*M* = 22.9, *SD* = 2.41) and high (*M* = 23.18, *SD* = 2.51, *p* = 0.79) levels of cognitive distraction. No other interaction effects that reached significance were observed. Note that there were fewer data points within this breathing range; thus, results should be treated with caution.

### 3.4. Olfactory Sensitivity and Weight Status

**Figure 5:**
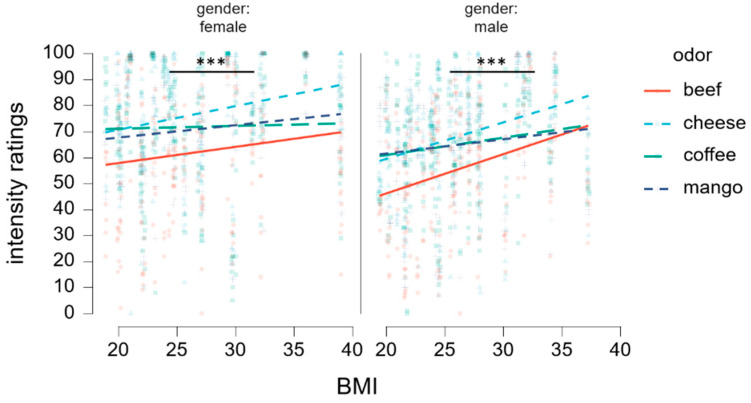
Positive correlation between the intensity ratings and the body mass index (BMI). Effects are presented separately across genders with each odor depicted by a different color. *** for *p* < 0.001.

Contrary to the common assumption that obesity is associated with reduced olfactory sensitivity, results of this study reveal the opposite effect. Specifically, BMI positively correlated with odor intensity ratings, highlighting higher sensory sensitivity in participants with an overweight or obesity status (*r* = 0.085, *p* = 0.017). Moreover, positive association between the intensity perception of odor stimuli and BMI was similarly expressed across genders (female: *r* = 0.128, *p* < 0.001; male: *r* = 0.210, *p* < 0.001) (see Figure 5). Interestingly, heightened sensitivity to the olfactory stimuli did not result in altered pleasantness perception as a function of BMI (female: *r* = −0.017, *p* = 0.696; male: *r* = −0.006, *p* = 0.579).

As a next step, we investigated how satiety levels might influence the perception of odors. Results of a correlation analysis revealed that intensity ratings decreased with rising satiety ratings (*r* = −0.056, *p* = 0.008), while pleasantness ratings were not affected by differences in satiety (*r* = 0.028, *p* = 0.189). On average, the difference between satiety ratings provided at the beginning (*M* = 32.78, *SD* = 27.80) and at the end of the experiment (*M* = 30.74, *SD* = 30.25) did not reach statistical significance (*p* = 0.416).

### 3.5. Physiological Data and Cognitive Distraction Paradigm

To ensure that the cognitive paradigm we used—the Tetris game—induced sufficiently different levels of cognitive distraction, we compared subjective ratings of perceived trial difficulty and participants´ game performance across trials assigned to either a low or high level of cognitive distraction (see Table 5).

To address a question potentially interesting to the audience, whether the odor modality could, in turn, influence the Tetris game performance, we conducted another exploratory analysis. The speculative idea behind this was that the coffee odor could elicit effects similar to caffeine intake, such as improving concentration and resulting in a higher Tetris score. More broadly, pleasantness perception could influence the game performance or the perception of difficulty, particularly when odors are either aversive or pleasant. To test this assumption, we computed two linear mixed models: one on the Tetris scores and one on difficulty ratings with fixed factor odor and random factor participants. Results indicated that the odor modality affected neither the Tetris score [*F* (4, 937.66) = 0.27, *p* = 0.893] nor difficulty perception of the Tetris game [*F* (4, 784.53) = 0.47, *p* = 0.755].

Yet, pleasantness perception interacted with the Tetris scores and perceived difficulty. Specifically, among female participants across both weight statuses, pleasantness ratings correlated positively with Tetris scores (*r* = 0.105, *p* < 0.001) and negatively with difficulty ratings (*r* = −0.067, *p* = 0.023). With regards to the weight status, participants in the obese group scored higher in the Tetris game when providing greater pleasantness ratings (*r* = 0.085, *p* = 0.005), and ratings of difficulty were correlated with a decrease in pleasantness ratings in the lean group (*r* = −0.063, *p* = 0.015). To conclude, we here observed effects of cognitive distraction on lean participants, while pleasantness correlated with the performance of females and participants with obesity. 

## 4. Discussion

Within the context of the widespread phenomenon of distracted eating, our study moves beyond the traditional focus on calorie intake to examine how cognitive distraction influences sensory perception. Considering the prominent role olfaction plays in eating behavior, we investigated the effects of cognitive distraction on the perception of food-associated odors. In our study, using a realistic and engaging cognitive distraction paradigm—the Tetris game—we explored how odor perception, in terms of pleasantness and intensity, changes under distraction for participants of different weight groups.

### 4.1. Modulation of Odor Perception by Gender and Weight Status under Cognitive Distraction

Our findings reveal that odor pleasantness is significantly diminished under high cognitive distraction. Noteworthily, the effects of distraction on odor pleasantness are modulated by weight status, in that pleasantness ratings decrease with a rise in cognitive distraction predominantly among lean participants. This differential response can be attributed to the distinct hedonic motivations and eating behaviors across study groups. On the one hand, individuals with overweight and obesity often exhibit heightened reward sensitivity and greater prominence of hedonic eating, prioritizing the anticipated pleasure from food over its immediate sensory qualities [42,43]. This hedonic motivation could dominate the focus and overshadow the effects of distraction, rendering subjects who are overweight less sensitive to changes in sensory pleasantness. On the other hand, the observed decrease in odor pleasantness during high cognitive load in lean individuals might act as a mechanism to prevent overeating by reducing the sensory reward value of food and lowering the likelihood of overconsumption when distracted. The opposite might be true as well: a decrease in pleasantness perception may promote overeating, to compensate for the reduced sensory satisfaction. Taken together, our results suggest that lean individuals may experience greater sensory disruption under distraction, potentially affecting appetite regulation, while those with obesity might maintain stable hedonic responses despite cognitive load. Although regular food consumption in distracted settings is among the largest predictors of heightened BMI, the complex interplay between body weight and susceptibility to distraction is yet to be understood.

With regards to intensity perception, in our study, we could not replicate prior findings demonstrating a decrease in odor intensity perception under distraction [19,20]. The difference in the outcome could not be attributed to the variations in the study design since the same Tetris paradigm was used to induce cognitive distraction [20]. Yet, it might be driven by the sensory properties of the stimuli utilized during the study. Specifically, findings from other studies across both taste and smell modalities suggest that the saliency of stimuli—primarily determined by their concentration levels or association with caloric density—appears to shape the distraction-induced effects. Notably, stimuli of high concentrations or those linked to high-caloric foods—like the odor stimuli used in this study (e.g., cheese, beef)—remained unaffected by distraction [15,18]. Additionally, there is a possibility that the level of distraction applied in our study was insufficient to impact the intensity perception of the study odors. This rationale is based on the observed trend of decreased intensity perception during the trials with elevated breathing rates, implying higher levels of difficulty and suggesting that a greater magnitude of distraction is required to affect intensity of the salient odor stimuli.

Beyond body weight, gender differences significantly contribute to variations in odor perception and eating behavior, orchestrated by distinct neurocognitive mechanisms. In obesity, food-related sensory cues have a stronger connection to reward-driven mechanisms among women, while eliciting higher activation in somatosensory regions in men [44,45]. In alignment with this, we observe an interaction between gender and effects of cognitive distraction. Specifically, under a high level of distraction, odor pleasantness significantly decreases in male, but not in female participants. One possible explanation is that cognitive distraction disrupts the integration of olfactory information in somatosensory regions, leading to reduced odor pleasantness in males. In contrast, due to being more strongly linked to reward processing, odor pleasantness perception in females remains intact, reflecting the resilience of reward pathways against distraction. Following this line of thought, our results show that females perform better in the Tetris game when exposed to the more pleasant odors. Again, this might be due to a close link between odors and emotional/reward processing, enhancing focus and cognitive efficiency [46]. Importantly, our results are not influenced by differences in the respiratory patterns, allowing us to assume that they are indeed induced by distraction and are modulated by interindividual and/or group differences. Furthermore, the absence of a significant difference in pleasantness ratings across both distraction conditions between lean and overweight/obese groups further supports the notion that the varying pleasantness ratings under distraction are not due to inherent differences in sensory perception.

### 4.2. Implications for Eating Behavior and Public Health

In broad terms, reduced pleasantness of odors might affect food-related behaviors, such as the acceptance of novel foods. When individuals are distracted, their reduced sensitivity to odor pleasantness may make them less likely to try and accept unfamiliar foods, as pleasant sensory experiences often facilitate the willingness to explore and accept new flavor options. Beyond food consumption, the decrease in odor pleasantness due to distraction may have broader implications for daily life experiences. Odors contribute to social interactions and emotional well-being, while enhancing mood and reducing stress [29,47,48]. The consistent reduction in odor pleasantness due to distraction could diminish these benefits and impact various aspects of quality of life, from healthcare to consumer experiences.

### 4.3. Differences in Odor Perception between Weight-Status Groups

In the context of research on sensory sensitivity and obesity, we report a notable positive correlation between intensity ratings and BMI. This discovery supports the findings of Stafford and Whittle [49] but diverges from the commonly reported evidence of diminished olfactory function in obesity [50]. Together, it underscores the complexity of this relationship as well as the current lack of agreement on it in the existing literature.

We also observed gender-driven differences in the context of olfactory sensitivity. Averaged across both distraction conditions, female participants provided significantly higher intensity ratings across all study odors compared to male participants, which resonates with prior suggestions of enhanced olfactory sensitivity among females [51].

Considering that a high level of cognitive distraction distinctly influences odor intensity and pleasantness perception in our study group, it is reasonable to assume two distinct distraction-related mechanisms through which the sensory-specific (intensity) and the affective (pleasantness) characteristics of odors are influenced. The distraction-induced evidence of another sensory modality, taste, supports this dichotomy [52].

### 4.4. Study Limitations and Future Directions

Despite the substantial contributions this work offers, several limitations warrant consideration. Our effects, though limited in size, align with prior research in the field reporting statistically significant yet relatively minor changes in olfactory and gustatory perception under distraction, reflecting the substantial interindividual differences characteristic of the chemical senses [19,20,22,52]. Furthermore, combining participants with an overweight status and those with obesity into a single group poses a limitation to our study. Future research should aim to differentiate between these groups to better understand the nuanced impacts of cognitive distraction on sensory perception. The sample size of 59 participants, while common for this field and sufficient for our study aims, should be considered a limitation when generalizing the results. Additionally, this study can be considered exploratory, as a formal sample size calculation was not performed, although the study design had been successfully tested before.

Another open question that remains is whether the decrease in odor pleasantness ratings we have observed is sufficient to induce altered food intake. Since neither intake nor food choice behavior was investigated in this study, our assumptions are purely based on the available knowledge from different research fields. Given the cross-sectional nature of our study, we cannot establish causal relationships between distracted sensory perception and overconsumption.

Future studies should directly bridge the changes in sensory perception to the immediate and delayed consumption. Moreover, due to our limited understanding of the neural mechanisms orchestrating distraction-induced changes observed behaviorally, especially with regards to the weight status and eating strategy individuals opt for, there is a clear need to investigate this in the future. Exploring eating behavior and individual differences as well as manipulating the saliency of sensory stimuli could provide further insights into why certain individuals are more susceptible to the effects of distraction.

## 5. Conclusions

In summary, our study provides novel insights into how cognitive distraction affects the intensity and pleasantness perception of odors, emphasizing the roles of weight status and gender. We found that distraction significantly reduces odor pleasantness perception, with this effect varying according to gender and weight status. By addressing the limitations of prior studies, we highlight the importance of considering these individual differences to develop more personalized and effective strategies for mitigating the negative consequences of distracted eating.

## Figures and Tables

**Table 1:** Characteristics of the study sample.

Characteristic	Total Sample (*n* = 59)	Lean Group (*n* = 30)	Overweight/Obese Group (*n* = 29)	*p*-Value
Gender				
Male	31	15	16	
Female	28	15	13	
Age (years)				
Mean (*SD*)	27.7 (6.56)	26.7 (6.4)	28.79 (6.72)	0.21
Range	19–48	19–43	19–48	
BMI (kg/m^2^)				
Mean (*SD*)	26.0 (4.5)	22.2 (1.7)	30.24 (2.58)	<0.001
Range	19.5–39.0	19.5–24.8	25.0–39.0	
SSI				
Mean (*SD*)	13.13 (1.4)	13.13 (1.23)	12.96 (1.58)	0.68
Range	11–16	11–16	11–16	
BDI				
Mean (*SD*)	5.31 (5.16)	4.3 (4.02)	6.3 (6.05)	0.148
Range	0–19	0–19	0–15	

**Table 2:** Cognitive distraction effects on pleasantness perception.

Pleasantness Ratings	Low Distraction (Mean ± *SD*)	High Distraction (Mean ± *SD*)	Estimate	Standard Error (*SE*)	95% Confidence Interval (*CI*)	*p*-Value
Overall Effect	46.17 ± 31.03	44.19 ± 30.01	1.98	0.91	[0.14, 3.74]	0.007
Group						
Lean	45.46 ± 28.76	43.76 ± 27.22	1.95	0.91	[0.14, 3.74]	0.035
Overweight/Obese	45.13 ± 33.37	43.29 ± 32.08	1.67	0.93	[−0.16, 3.51]	0.064
Gender						
Male	48.63 ± 29.14	45.33 ± 27.51	3.37	0.90	[1.60, 5.14]	<0.001
Female	41.03 ± 32.97	41.28 ± 32.17	0.24	0.95	[−1.62, 2.11]	0.791

**Table 3:** Pleasantness ratings across odor modalities.

Odor	Mean	Standard Deviation	*p*-Value
beef	34.62	24.03	<0.001
coffee	52.50	28.98	<0.001
mango	71.96	21.83	<0.001
cheese	21.44	20.59	<0.001
odorless	44.23	18.21	<0.001

**Table 4:** Intensity ratings across odor modalities.

Odor	Mean	Standard Deviation	*p*-Value
beef	58.35	24.06	<0.001
coffee	68.13	21.88	1.000
mango	67.55	20.95	0.975.
cheese	71.96	22.37	<0.05
odorless	18.11	20.52	<0.001

**Table 5:** Proof of the cognitive distraction paradigm.

Measure	Mean (*M*)	Standard Deviation (*SD*)	*F* Value	*p* Value
Tetris score			*F* (1, 48.66) = 64.28	<0.001
Low Distraction	1.95	1.54		
High Distraction	1.01	1.95		
Perceived difficulty			*F* (1, 50.17) = 279.63	<0.001
Low Distraction	20.35	20.58		
High Distraction	64.99	22.36		
Skin conductance			*F* (1, 55.98) = 0.44	0.505
Low Distraction	37.47	14.21		
High Distraction	34.36	11.48		

## Data Availability

The complete dataset generated and analyzed during the current study is publicly available (https://zenodo.org/records/12783330 accessed on 19 July 2024) [53].
